# Acupuncture and moxibustion for stress-related disorders

**DOI:** 10.1186/1751-0759-8-7

**Published:** 2014-01-24

**Authors:** Tetsuya Kondo, Masazumi Kawamoto

**Affiliations:** 1 Kansai University of Health Sciences, 2-11-1, Wakaba, Kumatori-cho, Sennan-gun 590-0482, Japan

**Keywords:** Acupuncture, Moxibustion, Stress, Fibromyalgia, Functional gastrointestinal disorder, Irritable bowel syndrome, Autonomic nervous system, Hypothalamo-pituitary adrenal axis, Sawada-style holistic therapy

## Abstract

Acupuncture and moxibustion, which medical doctors are licensed by the government of Japan to perform, can improve the psychological relationship between doctors and patients, especially when it is disturbed by a “game”, a dysfunctional interpersonal interaction that is repeated unintentionally. This advantage is due to the essential properties of acupuncture and moxibustion. Acupuncture and moxibustion are helpful in treating somatoform disorders, especially musculoskeletal symptoms. In Japan, a holistic acupuncture and moxibustion therapy called Sawada-style has been developed. This is based on fundamental meridian points that are considered to have effects on central, autonomic nervous, immune, metabolic, and endocrine systems to regulate the whole body balance. In addition, some of the fundamental points have effects on Qi, blood, and water patterns associated with major depression, generalized anxiety disorder, eating disorders, and somatoform disorders. The fixed protocol of Sawada-style would be suitable for large-scale, randomized, controlled studies in the future. Recent systematic reviews indicate that electroacupuncture would be a useful addition to antidepressant therapy for some symptoms accompanying fibromyalgia. Acupuncture and moxibustion are also recommended for irritable bowel syndrome, instead of Western drug therapy. Surprisingly, the dorsal prefrontal cerebral cortex, which is associated with a method of scalp acupuncture applied for gastrointestinal disorders, has been found to be activated in patients with irritable bowel syndrome. It is quite possible that regulation of this cortical area is related to the effect of scalp acupuncture. This acupuncture method can be effective not only for irritable bowel syndrome but also for other stress-related gastrointestinal disorders.

## Introduction

If a general practice doctor rules out typical disorders and fails to make a clinical decision in the case of a patient with a chronic complaint, the patient tends to be labeled as having an indefinite complaint and is advised to consult a psychiatrist on the grounds that the symptoms are just imaginary. The Japanese word meaning “imagination” is “Qi-no-sei”, which can also be translated as “because of Qi”. In order to treat such a patient, supplying or regulating Qi by acupuncture would be effective.

Table 1 shows the causes of each of five visceral dysfunctions according to traditional Chinese theory. As shown, as many as three of five viscera are vulnerable to psychological stress. Therefore, oriental medicine has an affinity to psychosomatic medicine.

**Table 1:** The causes of dysfunctions of the five viscera according to traditional Chinese theory

**Cause of dysfunction**	**Heart**	**Liver**	**Spleen**	**Lung**	**Kidney**
Aging	○				○
Psychological stress	○	○	○		
Chronic dysfunction of other organs	○	○		○	○
Infection		○		○	
Disregard for health		○	○		○
Environmental stress		○	○	○	○
Congenital		○		○	○

This review discusses (1) the advantages of the use of acupuncture and moxibustion by psychosomatic medical doctors, (2) The specific effects of acupuncture and moxibustion on musculoskeletal symptoms, (3) Japanese-style holistic acupuncture and moxibustion, to harmonize the whole body with regard to the mechanism of action, and (4) The effectiveness of acupuncture for fibromyalgia [1], chronic pain [1], and psychosomatic gastrointestinal disorders [2].

## Improvement of the psychological relationship between the patient and the therapist under the Japanese Medical Administration System for acupuncture and moxibustion

In China and Korea, the medical license for oriental medicine is completely independent of that for occidental medicine. In the same way, a license for acupuncturists and moxa-cauterizers is issued to those who pass a national examination in Japan. However, acupuncture and moxibustion are also permitted to all Japanese medical doctors, without the need for the specialist license. This Japanese system has many advantages. It is an advantage of the Japanese medical service system that medical doctors can choose between nerve block and acupuncture according to the condition of the patient [3]. In addition, physical contact through acupuncture or moxibustion can establish rapport if the psychological interview is difficult due to alexithymia or negativism. In Japan, a needle is inserted with a fine tube as a guide for the needle. A Japanese acupuncturist, Sugiyama, developed this technique. Since a tube surrounds the point of insertion, there is little insertion pain. This can soften the resistance to acupuncture, even by patients with excessive anxiety, and it helps establish and maintain confidence in the relationship between the patient and the therapist.

Nakamura et al. reported in a factor analysis of 197 patients that the attentive attitude of the therapist and proper touching by the therapist were significantly associated with a decrease in the subjective symptoms by acupuncture and moxibustion therapy, while the physical factors of the therapeutic stimulation were not [4]. These results indicate that acupuncture and moxibustion therapy is a kind of psychotherapy, which may be of assistance in establishing the psychological therapist-patient relationship, rather than a physical therapy.

According to transactional analysis theory, the therapeutic structure of acupuncture and moxibustion itself is sometimes helpful to escape from a “game” between the patient and the therapist. This is an acupuncture-specific effect and not the case with Kampo medicine. I previously reported that acupuncture broke down a “game” in the treatment of a patient with fibromyalgia who had been treated for 10 years and who had been unable to work for more than 12 hours per week [5]. The therapy had been disturbed by a hyperreactive action of the patient against the minimal touch by the acupuncturist, due to systemic hyperalgesia. A “yes, but” game was found between the patient and therapists. Searching for tender points, which is necessary and essential for acupuncture therapy, was of assistance in escaping from this “game” and enabled therapy for symptoms such as general fatigue. As a result, the patient acquired the ability to work for 23 hours per week, in addition to relief of pressure pain. This suggests that acupuncture therapy is suited to the treatment of patients with chronic, marked pain.

## Musculoskeletal symptoms

Knowledge of acupuncture and moxibustion is especially helpful for treating musculoskeletal symptoms, which are often complained of by patients with somatoform disorders, as mentioned below. The principle of acupuncture therapy depends on the three dimensional location of the complaint, since meridian points are selected from among the points that belong to the meridian that passes through the location of the symptom. This is also an essential difference between acupuncture and Kampo medicine, which is administered to the whole body, although a few Kampo formulas, such as formulas tonifying the kidney, tend to have an effect on lower energizer. The correspondence between the location of the symptoms and meridian points is much more complicated, since as many as twelve main meridians and six extra meridians run longitudinally, except for the belt vessel, and cross each other at crossing points.

**Figure 1:**
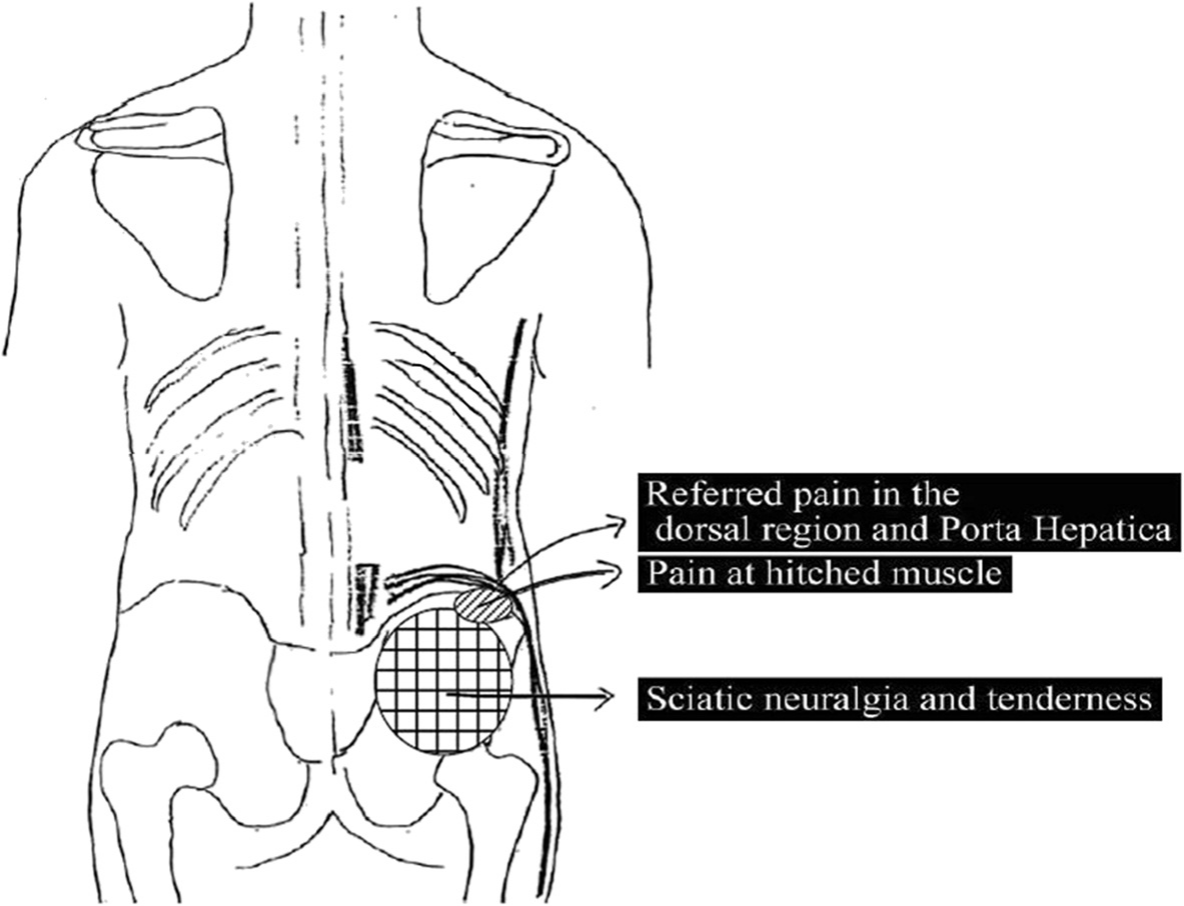
Chronic dorsalgia in a patient admitted with chronic pain disorder.

**Figure 2:**
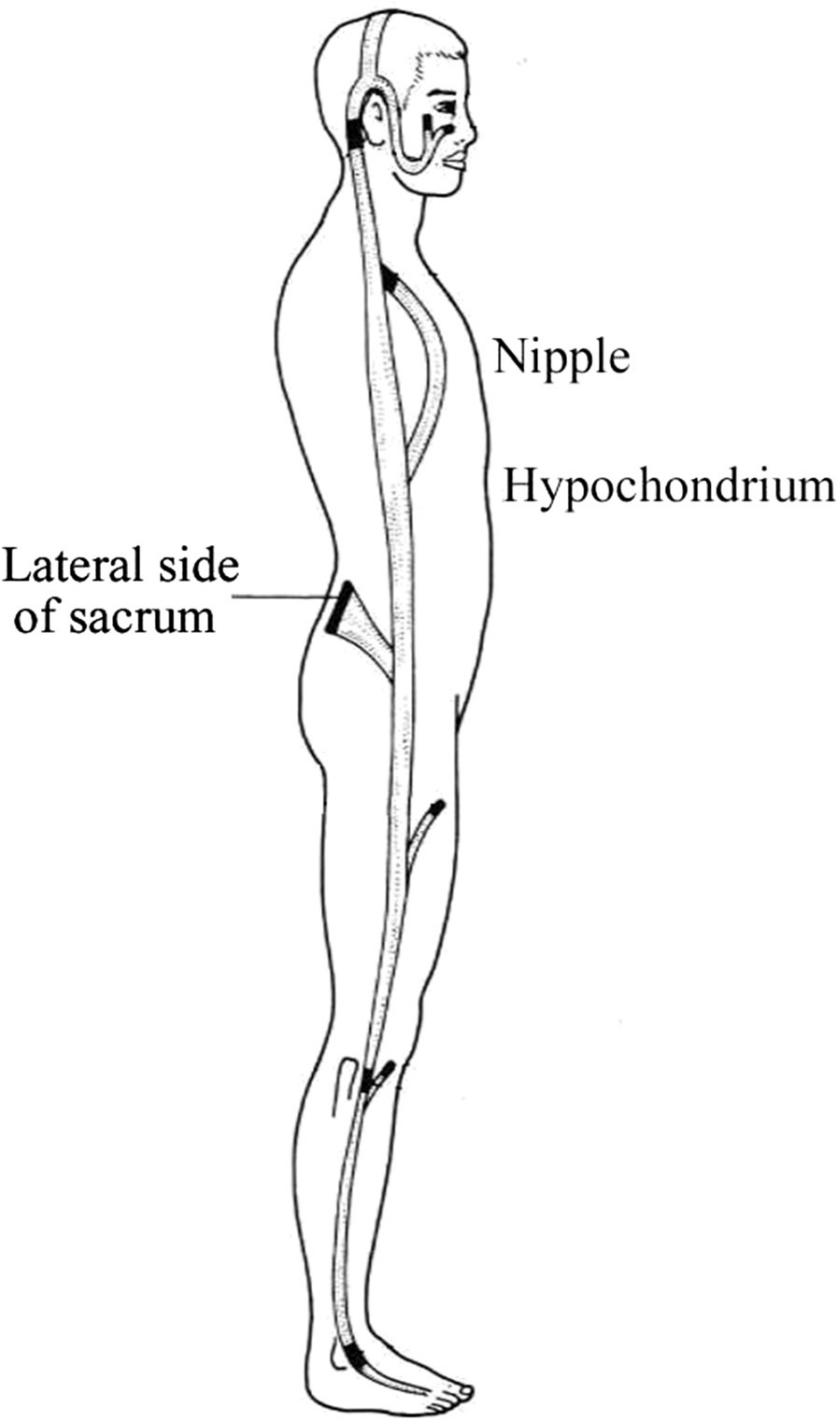
**Gallbladder ching muscle.** A modification of Irie’s illustration [6].

The case of a patient who was admitted with characteristic chronic lower dorsalgia, along with a feeling of tightness around the nipples, is shown in Figure 1. This patient had been diagnosed as having a chronic pain disorder and was under nonspecific treatment for chronic pain disorder, such as cognitive behavioral therapy including reading on Morita therapy and autogenic training, for four months. However, acupuncture therapy, which is specific for these symptoms, would have been more effective, since the symptoms closely resembled the symptoms due to dysfunction of the gallbladder ching muscle. There are twelve ching muscle systems in the human body. All of these are superficial lines, which run longitudinally between the head and extremities and are mainly associated with relatively superficial musculoskeletal symptoms, unlike the normal meridians that run deeply and are related to the viscera and bowels. Although each ching muscle runs along the corresponding main meridian, it merely indicates that multiple kinks tend to appear along the line, like a fictional, long, single muscle that has kinks. As shown in Figure 2, the gallbladder ching muscle runs from the third crural finger and ascends along the lateral side of the whole body. On the way, its branch lines adhere to bones or skin structures such as the nipples [6]. The symptoms due to dysfunction of this route closely resemble the complaints shown in Figure 1, and the kinks of the alternative lines across the nipples in Figure 2 may explain what the patient complained of as tightness around the nipples.

## Acupuncture and moxibustion for systemic regulation of the autonomic nervous, central nervous, immune, and endocrine systems

### The history of holistic therapy

Holistic therapy means treating the root by harmonizing the five viscera and six bowels. At the beginning of the 20th century, a Japanese acupuncturist, Sawada, developed the first holistic therapy, which is called “Sawada-style holistic therapy”. This therapy consists of moxibustion on the eleven fundamental meridian points that regulate the whole body and that are essential for every patient and additional points for the individual patient’s status [7].

The disorders susceptible to this therapy include nervous exhaustion and disorders for which occidental medicine is not sufficiently effective, such as daytime somnolence, tinnitus, and nocturnal perspiration. In China, acupuncture was virtually abolished in 1822 and remained disregarded until 1954. During this period, the Sawada-style was introduced to China and helped Chinese acupuncture continue its existence [8]. The essence of Sawada-style was published by Sawada’s disciple, Shirota [9,10]. This book functions as a bible for Japanese acupuncturists and moxa-cauterizers [11]. It is possible that this therapy involves the central nervous system. Unfortunately, no direct evidence for Sawada-style holistic therapy has been published.

Apart from Sawada-style holistic therapy, Kurono developed his own holistic therapy. He selected the fundamental meridian points according to the statistics on the previous frequency of his own usage, which are partially the same with those of the Sawada-style [12].

### The effects of fundamental meridian points for holistic therapies according to modern medicine

According to traditional Chinese theory, the 20 fundamental points for the Sawada-style or Kurono-style mentioned above have various physiological actions that are regarded according to modern medicine as actions on the central nervous, immune, endocrine, and metabolic systems, as shown in Table 2[13]. Shirota considered the principle of his holistic therapy, which was seven times of moxibustion per meridian point over the long term as a supplementary stimulation [9]. Kurono et al. employed acupuncture with relatively slight pressure, 20 g, which appeared to hardly raise habituation in a pilot study, while acupuncture with pressure of 60 g tended to raise habituation. Therefore, it would be useful to refer to reports on the physiological effects of supplementary acupuncture or moxibustion in understanding the mechanism of the action of holistic therapies. In the reports cited below, supplementary stimulations are mainly used such as electroacupuncture of 2 Hz [14,15], or 2–10 Hz [16].

**Table 2:** The effects of fundamental meridian points for the Sawada-style and Kurono-style holistic therapies on the central nervous, immune, endocrine, and metabolic systems

		**BL**	**BL**	**BL**	**BL**	**BL**	**BL**	**BL**	**BL**	**BL**	**CV**	**CV**	**GB**	**GB**	**GV**	**KI**	**LI**	**LR**	**ST**	**ST**	**TE**
		**10**	**11**	**13**	**14**	**18**	**20**	**23**	**25**	**32**	**12**	**6**	**20**	**21**	**12**	**6**	**11**	**14**	**25**	**36**	**4**
Sawada-style fundamental meridian points						○	○	○		○	○	○			○	○	○			○	○
Kurono-style fundamental meridian points		○	○	○	○		○	○	○		○	○	○	○				○	○		
Effects on the autonomic nervous system	Vagal nerve activity										↑									↑	
	Sympathetic nerve activity			↓										↑			↑				
	Heart rate									↑*											
Effects on the immune, endocrine and metabolic system	Anti-inflammatory effect		+	+		+	+ **	+			+		+			+	++ ***			+	+
	Reinforcing immunological resistance			+			+	+				+						+		+	
	Promoting oxygen intake and alimentation,and regulating body temperature							+	+		+	+						+			++
Effects on the central nervous system	Anxiolytic effect	+				+					++	+	+		++	++	+			+	
	Antidepressive effect					++					+		+	+				+		+	
	Analgesia	+	+		+				+				+	+			+		+	+	+

*Without effect on heart rate variability.

**On viscera.

***On cephalic and jugular area.

#### The effects of fundamental meridian points for holistic therapies on the autonomic nervous system

It was recently reported that some of these meridian points have effects on the autonomic nervous system. For example, CV12 [17] and ST36 [18] increase parasympathetic nerve activity. In addition, some of the meridian points have opposite effects, which are contrary to other points. LI11 [14] and GB21 [19] increase sympathetic nerve activity, while BL13 decreases it [20]. Since the point combinations in the holistic therapies include meridian points with bi-directional regulation of the autonomic balance, they can be applied to various pathological conditions in a balanced manner. Therefore, the holistic therapies can also be applied to chronic anxiety disorders [21] or severe depression [22,23], in which both sympathetic and parasympathetic nerve activities are reduced. BL32 reduces heart rate without an effect on heart rate variability [17], which might be effective for patients with palpitations without heart rate variability abnormalities.

#### The effects of fundamental meridian points for holistic therapies on the immune, endocrine, and metabolic systems

BL18, BL20, CV12, KI6, LI11, ST36, and TE4 have an anti-inflammatory effect on the immune system, while BL20, BL23, CV6, ST36, BL13, and LR14 reinforce the immune system according to traditional Chinese theory [13]. Stimulation of BL23, the dorsal transport point of the kidney meridian, promoted the secretion of adrenal steroid hormones [24,25]; this is quite reasonable, since the organ called the kidney in traditional Chinese theory includes the adrenal gland. If the hypothalamo-pituitary adrenal axis (HPA axis) is activated by acupuncture, cortisol is released, and this relieves the inflammation of arthritis or bronchial asthma. In fact, this meridian point has an anti-inflammatory effect on the five viscera according to traditional Chinese theory [9,14]. On the other hand, Toriizuka et al. reported that subcutaneous needles at BL23 increased norepinephrine and dopamine in the brain and prevented the decrease in the immune responses accompanying aging or menopause [26]. ST32 in addition to Extra37 also improved immune suppression after surgical stress [27].

Sawada laid weight on the triple energizer meridian, which was considered to promote oxygen intake and alimentation to regulate anabolism, catabolism, and body temperature [7,9,10,28]. Unfortunately, direct evidence that supports this theory was not found. The only evidence is a report that acupuncture including TE5 decreased vasomotor symptoms in cancer patients, probably due to raising the serotonin level to alter the body temperature set point [29].

#### The effects of fundamental meridian points for holistic therapies on mental status

BL10, BL18, CV6, CV12, GB20, GV12, KI6, LI11 and ST36 have an anxiolytic effect according to traditional Chinese theory [13]. There is evidence for some of these. For example, acupuncture for ST36 decreased anxiety-related behavior, the serum corticosterone level, and tyrosine hydroxylase-immunoreactive expression of rats under immobilization stress [15].

BL18, CV12, ST36, GB20, GB21 LR14, and ST36 have an antidepressive effect according to traditional Chinese theory. Among these meridian points, electroacupuncture at ST36 and GB20 reduced the Beck Depression Inventory scales in subjects with psychosomatic or psychiatric disorders such as fibromyalgia, irritable bowel syndrome, chronic fatigue syndrome, primary insomnia, and obsessive-compulsive disorder, probably by enhancing the intracephalic release of serotonin [16].

#### The effects of fundamental meridian points for holistic therapies on nociception

The fundamental points for holistic therapies include such meridian points as BL10, BL11, BL14, BL25, GB20, GB21, LI11, ST25, ST36 and TE4, which are also used for acupuncture analgesia, shown as “analgesia” in Table 2[30]. It has been reported that midbrain monoamines, especially serotonin and norepinephrine, are involved in acupuncture analgesia, not for surgical procedures but for the treatment of chronic pain [31]. There have been many reports indicating that these monoamine neurotransmitters released from the descending inhibition systems are involved in the effects of these meridian points. Increases in the synthesis and utilization of serotonin during acupuncture are supported by a double-blind study that showed that acupuncture analgesia was facilitated in patients who had been given a serotonin reuptake inhibitor [32]. This indicates that acupuncture has a synergistic action with serotonin reuptake inhibitors, which are commonly administered for psychosomatic or psychiatric disorders such as pain disorders and functional gastrointestinal disorders with a decreased visceral pain threshold or depression. In fact, Zhang et al. reported that a combination of weekly electroacupuncture for six weeks and paroxetine provided more effective treatment for depression than paroxetine alone [33]. The stimulation method is different between Sawada-style and acupuncture analgesia. Moreover, it is unclear whether Sawada selected the fundamental points in consideration of these detailed physiological effects. However, it is possible that the brain monoamine system is facilitated by holistic therapies, since Shirota reported that the Sawada-style was effective in treating many cases with depression or obsessive-compulsive disorder [10], for which monoamine reuptake inhibitors are given.

#### The effects of fundamental meridian points for holistic therapies on the mind-body correlation

Apart from the central nervous system, acupuncture at ST25 and ST37 relieved chronic visceral hypersensitivity in rats with irritable bowel syndrome [34]. In this study, changes in serotonin metabolism in the colon tissue were observed. ST25 also prevented chronic stress-induced increases in the sympathetic peptide, neuropeptide Y [35].

### The effects of fundamental meridian points for holistic therapies according to Qi, blood, and water theory

In Japan, an original scoring system was developed by Terasawa et al., which makes quantification of patterns possible (Table 3) [36,37]. This is quite helpful in clinical diagnosis and research, which is not the case in other countries. In this system, the scores for Qi deficiency, Qi stagnation, Qi counterflow, blood deficiency, blood stagnation, and water retention can be calculated. It is characteristic of Japanese oriental medicine to place weight on water retention instead of phlegm. Phlegm is generated when stagnant water loses its ability to flow and is more serous than water retention. This difference in the main pattern may be attributed to the differences in the climate between the dry continental climate of China and the wet monsoon climate of Japan [38].

**Table 3:** Terasawa’s Qi, blood, and water scoring system

**Qi deficiency**		**Qi stagnation**		**Qi flowback**		**Blood deficiency**		**Water retention**	
**Symptom**	**Score**	**Symptom**	**Score**	**Symptom**	**Score**	**Symptom**	**Score**	**Symptom**	**Score**
General fatigue	10	Depressive mood	18	Cold constitution and hot flush	14	Retardation of thought	6	Heaviness of body	3
Hypobulia	10	Heavy-headed feeling	8	Palpitation attack	8	Early-morning awakening	6	Systaltic headache	4
Easy fatigability	10	Unpleasant sensation of laryngopharynx	12	Sporadic headache	8	Asthenopia	12	Heavy-headed feeling	3
Hypersomnia	6	Chest oppression	8	Vomit	8	Dizziness	8	Carsickness	5
Anorexia	4	Hypochondrial oppression	8	Cough	10	Cramp	10	Dizziness	5
Vulnerability to cold	8	Abdominal fullness	8	Abrupt abdominal pain	6	Slight menorrhea	6	Dizzy feeling on standing up	5
Scariness	4	Counterchanging of symptoms	8	Scariness	6	Pale complexion	10	Watery rhinorrhea	3
Objective hollow voice	6	Difficulty in uprising	8	Impatience	8	Alopecia	8	Ptyalism	3
Pale tongue	8	Abdominal wind	6	Flush	10	Xeroderma	14	Foamy sputum	4
Vacuous pulse	8	Burping	4	Palpations above the umbilicus	14	Ungual incisure	8	Nausea	3
Flaccid abdomen	8	Sense of residual urine	4	Crural chills	4	Paresthesia	6	Rugitus	3
Uterine prolapse	10	Tympanicity	8	Palmar and plantar diaphoresis	4	Rectus abdominis spasm	6	Arthral tightening	7
Lower abdominal numbness	6							Edema	15
Diarrhea	4							Effusion	15
								Palpations above the umbilicus	5
								Watery diarrhea	5
								Oliguria	7
								Polyuria	5

For example, a diagnostic criterion, the Qi-deficiency score, was devised as follows. First, the author Terasawa observed the patients’ subjective symptoms and objective findings to achieve synthesis. The severity of Qi-deficiency, assessed by observation, was graded into four levels as an “overall scale” based on the author’s empirical knowledge. Second, the prevalence of the symptoms and signs related to Qi-deficiency in previous studies was ordered into four levels. The weight assigned to each symptom or sign with respect to the overall scale was estimated by multiple regression analysis, and this was used to create the Qi-deficiency score. In addition, Qi-stagnation, Qi-flowback, blood deficiency, blood stagnation, and water retention scores are calculated according to this system [37].

The fundamental meridian points for holistic therapies consist of meridian points with effects on these six Qi, blood, and water patterns in a balanced manner. This is why it is called a holistic therapy. These effects, in addition to their effects on yin deficiency patterns, and the corresponding therapeutic principles are listed in Table 4.

**Table 4:** The effects of fundamental meridian points for holistic therapies on Qi, blood, and water patterns

**Pattern**	**Related disorder**	**Therapeutic principle**	**BL**	**BL**	**BL**	**BL**	**BL**	**BL**	**BL**	**BL**	**BL**	**CV**	**CV**	**GB**	**GB**	**GV**	**KI**	**LI**	**LR**	**ST**	**ST**	**TE**
			**10**	**11**	**13**	**14**	**18**	**20**	**23**	**25**	**32**	**12**	**6**	**20**	**21**	**12**	**6**	**11**	**14**	**25**	**36**	**4**
Qi deficiency	Mood disorders and major depression	Tonifying Qi			+			+	+				+			+			+		+	
Qi stagnation		Soothing the liver and regulating Qi			+	+	+				+	+	+						+		+	
Qi counterflow	Anxiety disorders, generalized anxiety disorder and somatoform disorder in females	Downbearing Qi counterflow				+		+				+				+		+	+		+	
Blood deficiency	Generalized anxiety disorder in males and somatoform disorder in females	Tonifying blood					+	+													+	
Blood stagnation		Activating blood				+					+	+		+	+			+	+	+		
Water retention	Eating disorder in females	Dispelling edema							+						+		+	+		+	++	+
		Diuresis						+	+								+				+	
Yin deficiency		Tonifying yin					+		+								++					+

According to this system, the patterns associated with psychiatric and psychosomatic disorders were investigated in detail, in which Qi stagnation scores were associated with mood disorders and major depression [39]. This result is consistent with the notion that “Qi stagnation” consists of depressive mood, loss of interest, heavy-headed feeling, unpleasant sensation of the laryngopharynx, circadian rhythm of the symptoms, burping, and abdominal gas [36], which corresponds to the masked depression or “somatic anxiety” described in Hamilton’s rating scale for depression. In both sexes, high Qi deficiency scores were also associated with mood disorders and major depression and inversely associated with depressive disorder not otherwise specified [39]. For women, high Qi counterflow scores were associated with anxiety disorders, generalized anxiety disorder, and somatoform disorders [39]. On the other hand, high blood deficiency scores were associated with generalized anxiety disorder in men [39]. For women, high water retention scores were associated with eating disorders [39]. High Qi counterflow and blood deficiency scores for women and low water retention scores for both sexes were associated with somatoform disorders [39]. The associations of these disorders with the Qi, blood, and water patterns are also listed in Table 4.

Although Terasawa established no scoring system, yin deficiency listed in Table 4 is quite important. We previously reported three patterns of the depressive state, based on principal component analysis of the results of four examinations given to outpatients [40]. Of the three patterns, two yin deficiency patterns were accompanied by suicidal feelings or planning of suicide, while the depressive state of Qi deficiency pattern was not. This indicates that subjects with the heart yin deficiency-type depressive state are at risk of committing suicide. In this pattern, subjective and objective irritability, which are characteristic of heart yin deficiency, were considered associated with suicidal feelings or the planning of suicide.

In summary, the holistic therapies have physiological effects that correspond to therapeutic principles for mood, anxiety, somatoform, and eating disorders. In fact, a Japanese acupuncturist [41] reported a patient with a specific phobia treated with Sawada-style meridian points. At pain clinics, the Sawada-style is often used to treat chronic pain, which is often accompanied by indefinite complaints [3]. Regretfully, the Sawada-style has seldom been introduced abroad, and the reports mentioned below are not on the Sawada-style. However, its fixed protocol would be suitable for large-scale, randomized, controlled studies in the future.

### The total effectiveness of holistic therapies

It is a principle of Sawada-style holistic therapy to use not acupuncture but moxibustion. This is consistent with a report that acupuncture shows quick effects, while moxibustion is recommended for cases for which acupuncture is not sufficiently effective [42].

A randomized controlled trial using holistic therapy and placebo acupuncture for lumbago was done [43]. In this study, use of the thirteen fundamental meridian points for Kurono-style holistic therapy alone was more effective than placebo acupuncture, and as effective as the combination of the fundamental meridian points and electroacupuncture at BL23 and BL40 which are often used for lumbago. These results indicate that holistic therapy alone has a sufficient effect on lumbago, probably owing to adjustment of the whole body. Since this study was of parallel design with placebo acupuncture combined with a crossover design, the non-specific effects of the holistic therapy or electroacupuncture deriving from insertion of needles into the derma including non-meridian points could not be excluded.

Ishigami et al. reported that the Kurono-style fundamental meridian points significantly decreased the indefinite complaints in combination with ST36, which is one of Sawada’s, not Kurono’s fundamental meridian points, and CV17 [44], although it was not a controlled study.

## Effectiveness of acupuncture on fibromyalgia

A meta-analysis found no statistically significant effect of acupuncture on fibromyalgia [45]. However, if electroacupuncture is distinguished from manual acupuncture, the result is different [46]. In particular, randomized, controlled studies using a combination of high and low frequencies for three weeks showed a statistically significant difference between real and sham acupuncture [47]. Since this study was a randomized controlled study with sham acupuncture, the non-specific physiological effects of the insertion of needles in the control group could be excluded in addition to psychological placebo effects. In addition, Ezzo et al. reported the synergistic effect of acupuncture and antidepressants on pain, depression, and insomnia [1].

## Effectiveness of acupuncture on functional gastrointestinal disorders

Hypersensitivity of most of the digestive organs, especially in depressive patients, is known to contribute to painful functional disorders, such as irritable esophagus, functional dyspepsia, biliary dyskinesia, and irritable bowel syndrome. Xing et al. have reported that acupuncture, but not sham acupuncture, significantly increased the threshold of the rectal sensation of gas, desire to defecate, and pain, as compared to a control [48]. Recently, a meta-analysis reported that acupuncture-moxibustion for irritable bowel syndrome is better than the conventional Western drug therapy [49].

Although the evidence level is lower than for irritable bowel syndrome, Xu et al. found that regular acupuncture had better therapeutic effects and fewer side effects in improving gastric motility and relieving discomfort sensations in functional dyspepsia compared to internationally accepted medicines such as cisapride and motilium [50].

Recently, activation of the right dorsal prefrontal area in patients with irritable bowel syndrome [51] and stress-induced visceral hyperalgesia [52] has been reported. Functional MRI has shown that activation of such regions as the prefrontal area is associated with a low visceral pain threshold in patients with irritable bowel syndrome [53]. The activation of this area and visceral pain was reduced by amitriptyline [54].

According to Jiao’s scalp acupuncture theory, stimulation of the scalp over the cerebral cortex has an effect on the functional localization corresponding to that of the cerebral cortex [55]. Actually, it was reported that scalp acupuncture in the forehead area changed the glucose metabolism in the cortex beneath [56]. Interestingly, the right dorsal prefrontal area is anatomically beneath Jiao’s “stomach area” and “intestinal area” on the scalp according to scalp acupuncture theory. Scalp acupuncture in these areas may influence the neural activity in the right dorsal prefrontal area. Therefore, it would be quite rational to stimulate this area as a therapy for irritable bowel syndrome. Although there have been few reports on scalp acupuncture for irritable bowel syndrome, a randomized controlled study in which the effects of scalp acupuncture were directly compared with those of western medication was done [57]. Neither a placebo effect nor non-specific physiological effect of the insertion of acupuncture needles in the control group could be excluded since neither a placebo nor sham control group was set. However, it was shown that scalp acupuncture in Jiao’s stomach area and intestinal area was significantly more effective for diarrhea-type irritable bowel syndrome than was western drug therapy.

## Conclusions

Acupuncture and moxibustion are helpful for improving the psychological relationship between the therapist and the patient, especially with respect to a negative “game”, and they are especially effective for chronic pain, fibromyalgia, irritable bowel syndrome, and functional dyspepsia, even if serotonin reuptake inhibitors have already been administered. Japanese Sawada-style holistic therapy can regulate the whole body with effects on brain monoamines and the autonomic nervous, immune, metabolic, and endocrine systems.

## Abbreviations

HPA axis: Hypothalamo-pituitary adrenal axis.

## Competing interests

The authors declare that they have no competing interests.

## Authors’ contributions

TK drafted the paper. MK revised the paper and provided books of reference. Both authors read and approved the final version.
